# Evaluation of the Choose Home Intervention to Reduce Health Services Use and Promote Aging in Place

**DOI:** 10.1093/geroni/igaf122.4339

**Published:** 2025-12-31

**Authors:** Daniel Harris, James Rudolph, Christopher Halladay, Malisa Barber, Alexandra Nothern, Jane Driver

**Affiliations:** University of Delaware, Newark, Delaware, United States; VA Providence, Providence, Rhode Island, United States; Providence VAMC THRIVE-COIN, Providence, Rhode Island, United States; Department of Veteran Affairs, Providence, Rhode Island, United States; VA Boston Healthcare System, Boston, Massachusetts, United States; VA Boston Healthcare System, Brockton, Massachusetts, United States

## Abstract

Nearly all older Veterans have expressed the desire to remain in their homes and to avoid the use of acute health services, yet complex comorbidity and limited resources at home represent key barriers towards achieving “aging in place.” To address gaps in the care continuum for older Veterans at-risk for acute health services use and to promote aging in place, VA Boston Healthcare System developed Choose Home (CH), an interdisciplinary team providing short-term, in-home medical and social care to stabilize veterans at risk of losing independence. Between 2023 and 2025, a total of 219 Veterans (mean age = 80.7 years; 85.8% White; 38.5% diagnosed with dementia) were admitted to CH. We used electronic health records to measure emergency department (ED) visits, inpatient admissions, and hospital length of stay before and after CH admission. Poisson regression with robust standard errors estimated incidence rate ratios (IRRs) and 95% confidence intervals (CIs) comparing health services in the 61 to 120 days before admission (reference) to 60-day periods following admission. Compared to the reference period, ED visits decreased by 40% (IRR=0.60, 95%CI=0.34-1.02) and 63% (IRR=0.37, 95%CI=0.20-0.69) in the 61-120 days and 121-180 days after admission, respectively. Hospital length of stay (121-180 days IRR=0.45, 95%CI=0.26-0.76) and inpatient admissions (121-180 days IRR=0.52, 95% CI=0.34-0.79) decreased by 55% and 48%, respectively. The CH program is a scalable intervention that appears to be effective at reducing acute health services use among older Veterans at increased risk of health-related complications.

